# Arctigenin Attenuates Tumor Metastasis Through Inhibiting Epithelial–Mesenchymal Transition in Hepatocellular Carcinoma *via* Suppressing GSK3β-Dependent Wnt/β-Catenin Signaling Pathway *In Vivo* and *In Vitro*

**DOI:** 10.3389/fphar.2019.00937

**Published:** 2019-08-29

**Authors:** Zheng Lu, Lingling Chang, Hongbo Zhou, Xiaoqiang Liu, Yinqian Li, Tiejun Mi, Dewen Tong

**Affiliations:** ^1^College of Veterinary Medicine, Northwest A&F University, Yangling, Shaanxi, China; ^2^College of Veterinary Medicine, Huazhong Agricultural University, Wuhan, China

**Keywords:** arctigenin, hepatocellular carcinoma, anti-metastasis, anti-epithelial – mesenchymal transition, Wnt/β-catenin

## Abstract

Arctigenin (ARG) has been reported to be a bioactive lignan from *Arctium lappa* exerting various activities including anti-cancer and immune-regulation. The present study aimed to investigate the anti-metastasis activity and mechanism of ARG against hepatocellular carcinoma *in vitro* and *in vivo*. The results showed that ARG exhibited a significant cytotoxicity on Hep G2 and SMMC 7721 cells (but not on normal liver cells LO2). In addition, the migration and invasion of Hep G2 and SMMC 7721 cells were also remarkably repressed. Furthermore, ARG attenuated Wnt/β-catenin signaling activation, resulting in the down-regulation of β-catenin target genes including c-Myc, cyclin D1, MMP-9, and ZO-1. Noticeably, ARG attenuated the activation of Wnt/β-catenin through a GSK3β-dependent pathway. Besides, we also found that ARG potentially inhibited epithelial–mesenchymal transition by up-regulating the epithelial and down-regulating the mesenchymal marker proteins. *In vivo*, intraperitoneal injection of ARG not only significantly inhibited the growth of subcutaneous transplanted tumor but also dramatically alleviated the tumor metastasis in liver. Our data demonstrated that ARG exerted anti-epithelial–mesenchymal transition and anti-metastasis activities against hepatocellular carcinoma, which might make it a candidate as a preventive agent for cancer metastasis.

## Introduction

Hepatocellular carcinoma (HCC) is one of the most common human malignancies; majority of the patients usually manifest a poor prognosis (Xie et al., 2019). Although early-stage and primary hepatic carcinoma can be treated by surgery, radiofrequency ablation, and transarterial chemoembolization, most cases eventually develop locally advanced or metastatic diseases, which cannot be treated using the aforementioned loco-regional therapies, and hence indicated for systemic therapy (Grandhi et al., 2016; Dutta and Mahato, 2017). According to the statistics, hepatocellular carcinoma is the second most common cause of cancer-related deaths in China and the third leading cause of death in the world (Omata et al., 2017). Accumulating evidences suggested that the high metastatic potential is the pivotal reason that caused the high mortality in malignancies including hepatocellular carcinoma (Mehlen and Puisieux, 2006). The treatment for metastatic cancer is to control the metastasis and improve patient survival, as opposed to curing the disease. Therefore, more and more attentions have been paid to develop novel anti-cancer agents targeting molecules involved in the development and maintenance of tumor metastasis (Wan et al., 2013; He et al., 2015).

It has been proven that epithelial–mesenchymal transition (EMT) plays a critical role in tumor metastasis (Li and Li, 2015). During the process of EMT, there are series of phenotype changes that occurred in tumor cells such as proliferation attenuation, cell–cell adhesion decrease, depolarization secretion increase, and motility augmentation (Santamaria et al., 2017). Changes in cell adhesion and polarity enable tumor cells to dissociate and migrate from the primary tumor and result in the tumor invasion, which is considered as an essential step in the metastatic cascade of carcinomas (Wan et al., 2013). Further investigations revealed that the up-regulation of mesenchymal markers (including N-cadherin, vimentin, and fibronectin), the down-regulation of epithelial markers (including E-cadherin and laminin), the altered expression of nuclear transcription factors (including Snail, Slug, ZEB1/2, and Twist1/2), and the rearrangement of cytoskeleton proteins (including occluding and ZO-1) are involved in tumor cells undergoing EMT (Peinado et al., 2007; Zhu et al., 2013). In recent years, plenty of natural compounds have been reported to exhibit an anti-metastasis effect *via* inhibiting EMT in malignancies. For example, several studies demonstrated that polyphenols including epicatechin, quercetin, carnosol, and flavonoids could predominantly reverse the metastasis in various malignancies by targeting EMT (Amawi et al., 2017).

The Wnt/β-catenin signal has been demonstrated to play an important role in tumor invasion and metastasis (Chen et al., 2017). There are several key components in the canonical Wnt/β-catenin singling pathway: Wnt, an extracellular ligand protein; Frizzled (Fz) and low-density lipoprotein receptor-related protein 5 or 6 (LRP5/6) located on cell membrane, as receptors; β-catenin, a cytoplasmic signaling transducing activator. Cytosolic β-catenin could not accumulate until Wnt combines with Frizzled, which is due to the destabilization and degradation mediated by a destruction complex composed of casein kinase 1a (CK1a), adenomatous polyposis coli (APC), Axin, and glycogen synthase kinase 3β (GSK3β) (Barker, 2008). Accumulated cytoplasmic β-catenin can be translocated into the nucleus where it binds to various transcription factors, resulting in the activation of its target genes including c-myc, cyclin D1, ZO-1, matrix metalloproteinases (MMP), and survivin (Angers and Moon, 2009; Zhan et al., 2017). Mutations of the canonical Wnt cascade genes and hyperactivation of the Wnt/β-catenin signaling pathway appear to be the most frequent genetic event and contribute to liver carcinogenesis and metastasis, even resistance to chemo-therapeutic agents (Pez et al., 2013; Yamashita and Wang, 2013).

**Figure 1:**
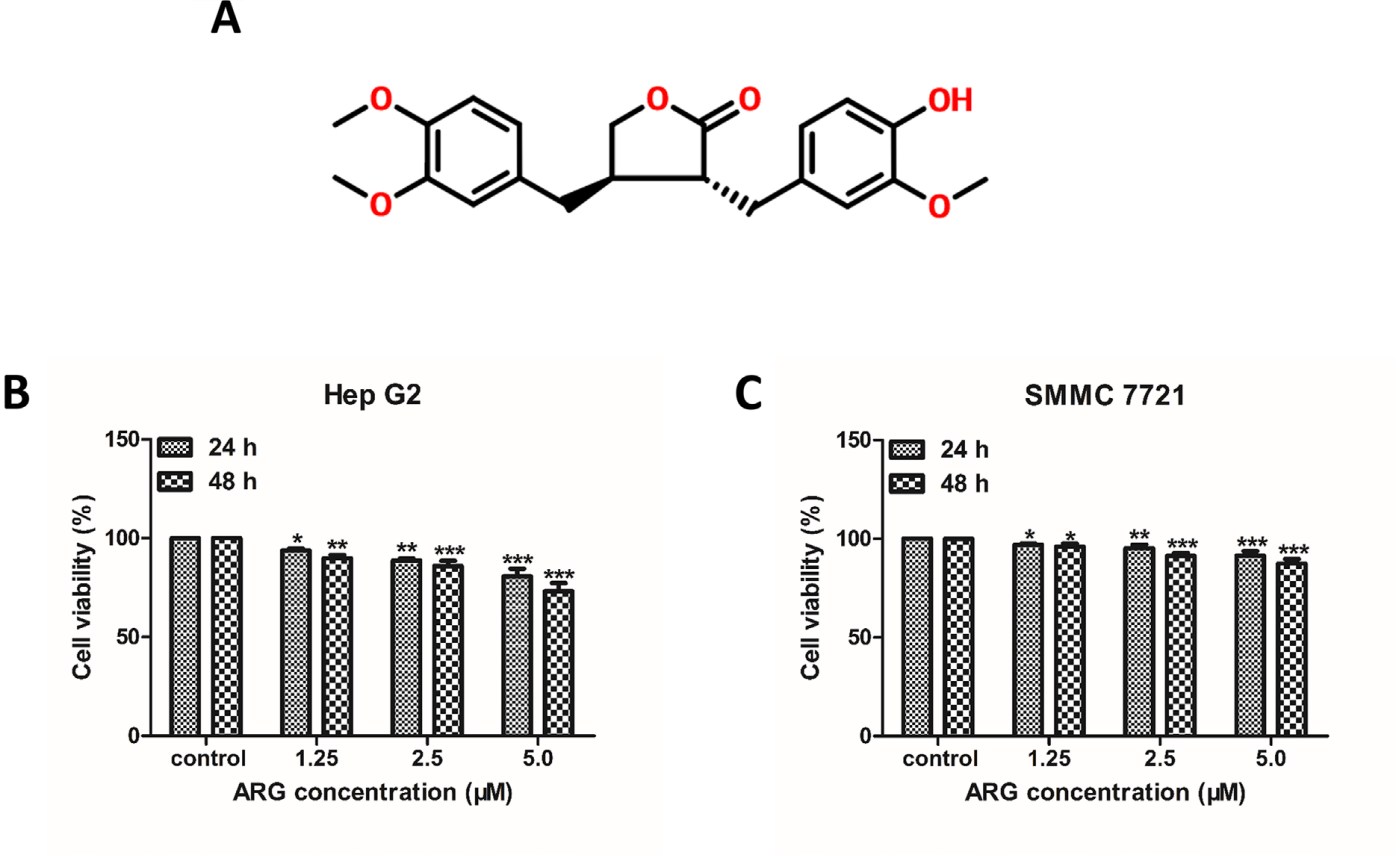
Arctigenin (ARG) inhibits the proliferation of human hepatoma carcinoma cells (HCC). **(A)** Chemical structure of ARG. **(B)** Hep G2 and **(C)** SMMC 7721 cell lines were incubated with ARG (0, 1.25, 2.5, 5.0 μM) for 24 and 48 h, respectively. Cell viability was determined by MTT assay. Results were presented as means ± SD, n = 3. *p < 0.05, **p < 0.01, ***p < 0.001 versus the control group.

Thus, development of new anti-cancer drugs targeting Wnt/β-catenin signal becomes a potential therapeutic intervention against HCC (Chen et al., 2017). There are numerous natural compounds that have been found inhibiting the tumor metastasis by modulating Wnt/β-catenin signal pathway, such as curcumin (Dou et al., 2017) and Destruxin B (Huynh et al., 2014). Obviously, pharmacological inhibition of Wnt/β-catenin pathway could lead to suppression of cell proliferation and increased sensitivity to some anti-cancer drugs. *Arctium lappa* is commonly known as a perennial economic crops in China and oriental countries. The rhizome of *Arctium lappa* is a kind of nutritious vegetable, and the seed has been traditionally used as herbal medicine. As we know, Arctigenin (ARG) (chemical structure shown in **Figure 1A**) has been reported to be a bioactive lignan from *Arctium lappa* and demonstrated to be effective in numerous pharmacological activities, including anti-cancer (Lu et al., 2015; Lee et al., 2017), immune-regulation (Lu et al., 2018), cardiovascular protection (Liu et al., 2015b; Daci et al., 2017), and anti-viral (Shen et al., 2018). We have previously proved that ARG could induce apoptosis in HCC including Hep G2 and SMMC7721 cells with mild cytotoxicity in normal hepatic cells (Lu et al., 2015). However, whether the anti-EMT and anti-metastasis were also involved in the anti-tumor activity of ARG in hepatocellular carcinoma still remained unclear. The present study aimed to investigate the anti-metastasis activity and mechanism of ARG against hepatocellular carcinoma *in vitro* and *in vivo*.

## Materials and Methods

### Reagents

RPMI 1640 medium and fetal bovine serum (FBS) were purchased from Life Technologies Inc. (Shanghai, China). Primary antibodies for immunofluorescence including rabbit monoclonal anti-β-catenin and mouse monoclonal anti-E-cadherin were bought from Abcam (Cambridge, UK). The secondary anti-rabbit and anti-mouse antibodies were bought from Boster Biological Technology (Wuhan, China). Rabbit polyclonal antibodies against p-GSK3β, N-cadherin, and E-cadherin; rabbit monoclonal antibodies against c-Myc and cyclin D1; and mouse monoclonal antibodies against vimentin and MMP-9 were purchased from Abcam (Cambridge, UK). Rabbit polyclonal antibodies against β-catenin and GSK3β were purchased from Proteintech (Wuhan, China). Rabbit polyclonal antibody against ZO-1 was bought from Bioss (Beijing, China). Rabbit polyclonal antibody against p-β-catenin and rabbit monoclonal antibodies against GAPDH, β-actin, and histone H3 were obtained from Cell Signaling Technology, Inc. (Danvers, MA, USA). All other chemicals and reagents used in this study were of highest quality and obtained from standard commercial sources.

Arctigenin (ARG) (purity≥98%) purchased from JCKY Institute of Chemical Technology (Beijing, China) was dissolved at 20 mM with DMSO as a stock solution and stored at −20°C. Before all experiments, the desired concentrations of ARG were freshly diluted with medium from the stock.

### Cell Culture

Human hepatoma carcinoma cell lines Hep G2 and SMMC 7721 and normal liver cell line LO2 purchased from ATCC were maintained in RPMI-1640 medium supplemented with 10% heat-inactivated FBS (v/v), 100 U ml^−1^ penicillin, and 100 μg ml^−1^ streptomycin. Cells were cultured at 37°C with 5% CO_2_.

### Measurement of Cell Viability

LO2, Hep G2, and SMMC 7721 cells (1×10^3^ cells) were seeded in a 96-well plate and incubated with medium containing the indicated concentrations of ARG (1.25, 2.5, 5.0 μM) for 24 and 48 h. After incubation, cells were incubated with 1 mg/ml MTT in complete growth medium at 37°C for 4 h. Then, the culture medium in each well was replaced by 100 ml DMSO to dissolve the formazan crystals generated by living cells. Absorbance at the wavelength of 490 nm was read in a M200 PRO Micro-plate Reader (TECAN, Switzerland).

### Wound Healing Assay

The effect of ARG on HCC migration was examined by a wound healing assay. Hep G2 and SMMC 7721 cells were seeded in 6-well plates and cultured for 24 h. An artificial wound was made by scratching the cell monolayers with a sterile 10 μl pipette tip. Then, the cells were incubated with the indicated medium. Five optical fields (10× magnification) were randomly chosen to record the wound areas at different time points (0, 24, and 48 h) using IX51 microscope (OLYMPUS, Japan). Cell migration was assessed by measuring gap sizes using Image J (National Institute of Mental Health, Bethesda, MD).

### Cell Invasion Assay

The effects of ARG on HCC invasion were determined using a Trans-well Boyden Chamber (BD Biosciences, USA). The upper chamber was coated with diluted matrigel (1 mg/ml). Hep G2 and SMMC 7721 cells were seeded in the upper chamber at a density of 2×10^4^ (100 μl per well), followed by the addition of indicated medium. The lower chamber contained 0.5 ml RPMI-1640 medium supplemented with 10% FBS. After 48 h incubation at 37°C, the migrated cells were fixed with 95% ethanol and stained with 0.5% crystal violet (Beyotime Biotech., Jiangsu, China) and subsequently rinsed twice with PBS. For the analysis, five random fields (10× magnification) of each well were photographed by IX51 microscope (OLYMPUS, Japan) and quantitatively analyzed by Image J software.

### Immunofluorescence

After treatment, Hep G2 and SMMC 7721 were fixed with 4% paraformaldehyde for 15 min, permeated with 0.5% Triton X-100 for 20 min, and blocked with 5% BSA for 1 h. The cells were washed with ice-cold PBS and incubated with primary antibodies rabbit anti-β-catenin (1:100) and rabbit anti-E-cadherin (1:100). Then, the cells were incubated with FITC-conjugated secondary antibodies for an additional 1 h. The nuclei were stained with DAPI for 5 min. Finally, cells were visualized using BX53 fluorescence microscope (OLYMPUS, Japan).

### Western Blotting and Co-Immunoprecipitation Analysis

After treatment, cells or tissues were lysed in RIPA buffer. The protein concentration was determined using a BCA protein assay kit (Thermo Scientific, USA) according to the manufacturer’s instructions. Whole cell lysates (40 μg protein) were resolved by 10% SDS-polyacrylamide gels electrophoresis and transferred onto PVDF membranes. Non-specific binding was blocked by Blocking Buffer (Beyotime Biotech., Jiangsu, China) for 3 h at 37°C. Subsequently, membranes were incubated with specific antibodies at an appropriate dilution overnight at 4°C. The membranes were washed with TBS with Tween-20 (TBS-T) and then incubated with HRP conjugated second antibodies. Specific complexes were visualized using Chemiluminescent HRP Substrate (Millipore Inc., MA, USA). Densitometry was performed using the software Image J. All data were obtained in triplicate, independent experiments.

For co-immunoprecipitation, 300–500 μg extracted protein were incubated with 1 µg of appropriate antibody and 10 μl of pre-rinsed protein-A/G-beads (Santa Cruz, CA) at 4°C overnight. The samples were centrifuged at 3,000 rpm for 5 min, the supernatant was discarded and rinsed with lysis buffer twice, and then 2×SDS loading buffer was added before analysis by immunoblotting.

### Quantitative Real-Time PCR

Total RNA was extracted using Trizol (Invitrogen, Shanghai, China) according to the manufacturer’s protocol and reverse-transcribed into cDNA using PrimeScript™ RT reagent Kit with gDNA Eraser (TaKaRa Bio Inc., Tokyo, Japan). Real-time PCR was performed in 96-well plates in a total reaction volume of 20 µl on Bio-Rad IQ5 Real-Time PCR System using SYBR^®^ Premix Ex Taq™ II (TaKaRa Bio Inc., Tokyo, Japan). The relative expression level of mRNAs was normalized to that of the internal control β-actin using the 2^−ΔΔCt^ cycle threshold method. All related gene sequences were listed in **Supplementary Table 1**. All experiments were independently performed three times, and the average was used for comparison.

### Mouse Xenograft Tumor Model and Metastatic Tumor Model

Mouse tumor model studies were carried out in accordance with the principles and procedures of the Institute Ethical Committee for Experimental Use of Animals. Athymic nu/nu mice (body weight 18–22 g) at the age between 6 and 8 weeks were kept in a SPF condition, feeding with sterile rat diet. Both mice (#43004700044086) and rat diet (#43004700058143) were purchased from Hunan SJA Laboratory Animal Co., Ltd (Hunan, China). Hep G2 cells (2×10^6^ in 0.2 ml physiological saline) were injected subcutaneously into the axillary fossa to build a xenograft tumor model. Subcutaneous tumors were allowed to grow for 10 days until the tumor size reached 50 mm^3^. Hep G2 cells (1×10^5^ in 0.1 ml physiological saline) were injected intravenously *via* the tail vein to build a metastatic tumor model. ARG was given at 10, 20, or 40 mg/kg in a clear solution containing 40% polyethylene glycol and 10% ethanol *via* intraperitoneal injection in a volume of 0.2 ml. The negative control group was treated with an equal volume of vehicle. Group of normal mice administrated with an equal volume of vehicle intraperitoneally once daily served as normal control. Body weight and tumor size were measured every 3 days. After 5 weeks, the subcutaneous tumors were removed, weighed, and fixed for IHC analysis. For tumor metastases analysis, mice were sacrificed, the organs including the brain, liver, lung, spleen, and kidney were excised, and the metastatic status was examined using HE staining analysis. Six sections from different parts of each organ were screened histologically, and the number of metastases nodules was counted for statistical analysis.

### H&E Staining

Tumors, kidneys, livers, lungs, brain, lymph gland, and spleens were collected, fixed in poly-formaldehyde fixed solution, and embedded in paraffin. Sections (4 μm) were stained with hematoxylin and eosin to facilitate histologic examination. Representative images (20× magnification) were taken on IX51 fluorescence microscope (OLYMPUS, Japan).

### Immunohistochemistry (IHC)

Xenograft tumor tissues were fixed in 4% paraformaldehyde and embedded in paraffin. Serial 4 μm histological sections were deparaffinized in xylene and rehydrated in graded concentrations of ethanol. Then, 3% H_2_O_2_ was used to quench the endogenous peroxidase. Subsequently, the sections were submitted to antigen retrieval and incubated with 5% BSA. After incubating with anti-β-catenin (1:50) or E-cadherin (1:100) antibodies overnight at 4°C, the sections were incubated with HRP-conjugated secondary antibodies for 30 min at room temperature. Finally, the peroxidase reaction was developed with 3,3′-diaminobenzidine and counterstained with hematoxylin. Immunostaining pattern for each case was independently analyzed by two pathologists using BX53 fluorescence microscope (OLYMPUS, Japan).

### Statistical Analysis

The statistical analysis was performed by one-way analysis of variance (ANOVA) using GraphPad prism 5.0 software by Dunnett’s test. Values are expressed as mean ± SD, and all experiments were performed at least in triplicate. Cases in which *p* < 0.05 were considered statistically significant.

## Results

### ARG Exerts Anti-Proliferative, Anti-Metastasis, and Anti-Invasion Effects on Human Hepatoma Carcinomacells (HCC)

**Figure 2:**
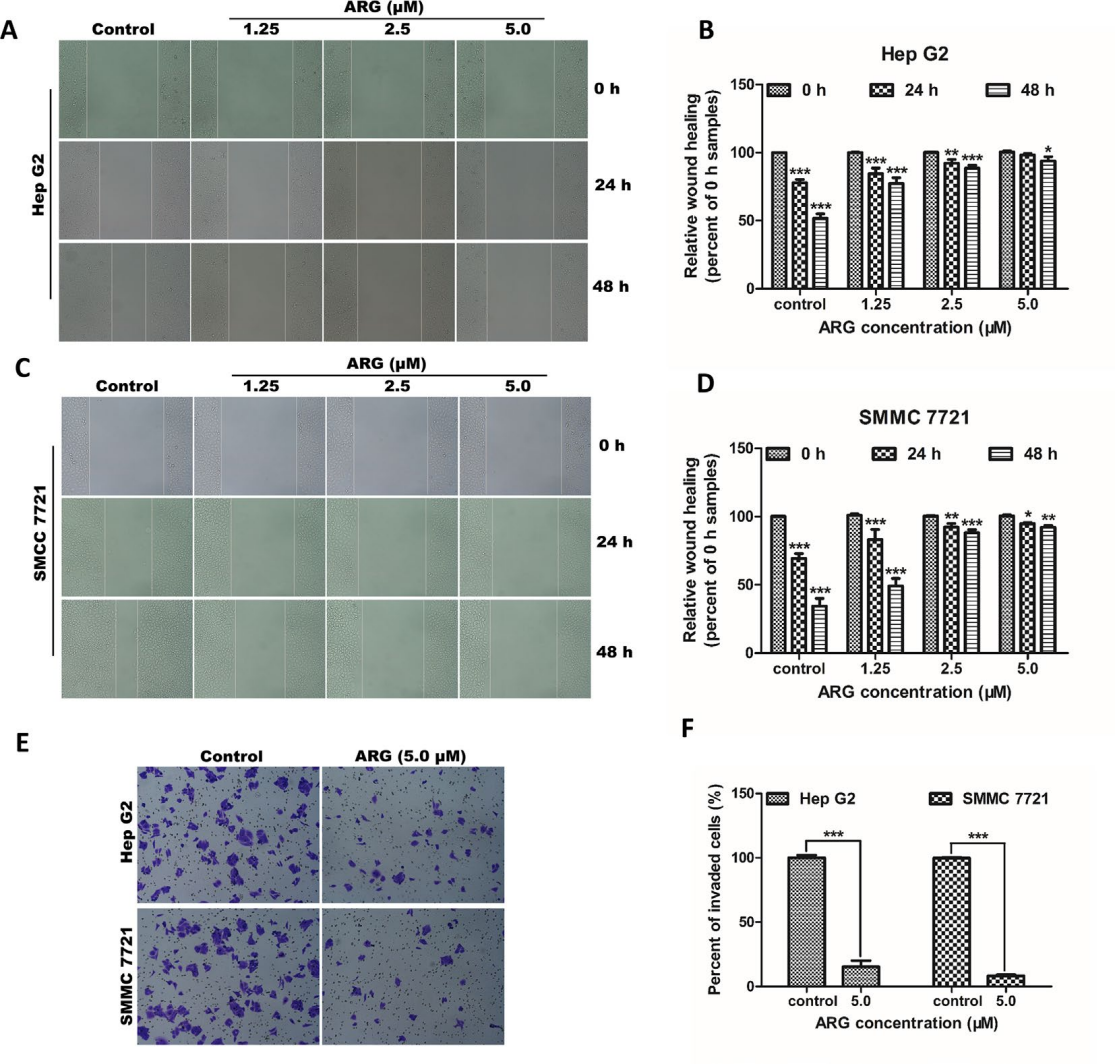
ARG exerts anti-metastasis and anti-invasion effects on HCC. **(A** and **C)** Representative images of the wound healing assay from Hep G2 and SMMC 7721 cells after indicated treatments (100× magnification). **(B** and **D)** Percentage of wound closure compared to the 0 h samples in Hep G2 and SMMC 7721 cells. **(E)** Representative images of the transwell invasion assay from Hep G2 and SMMC 7721 cells treated with ARG (5.0 μM) for 48 h (200× magnification). **(F)** Percentage of the invaded cells compared to the control samples. The experiments were done in triplicates, and results were presented as means ± SD, n = 3. *p < 0.05, **p < 0.01, ***p < 0.001 versus the control group.

To investigate the anti-proliferative effect of ARG on HCC, two HCC cell lines including Hep G2 and SMMC 7721 were chosen to perform MTT assay. As shown in **Figures 1B , C**, the cell viability of HCC was significantly suppressed by ARG in concentration- and time-dependent manner. But no cytotoxicity effect was found on normal liver cell line LO2 (**Supplementary Figure 1**). To further observe the anti-cancer effect of ARG, wound healing and trans-well assay were performed to assess the metastasis and invasiveness of HCC, respectively. Results in **Figures 2A–D** revealed that ARG potently inhibited the migration of both Hep G2 and SMMC 7721 cells in a concentration- and time-dependent manner. Additionally, treatment with ARG obviously suppressed the invasiveness of Hep G2 and SMMC 7721 cells to pass through the matrigel and membrane barriers in the trans-well (**Figures 2E, F**). All these results demonstrated that ARG exerted not only an anti-proliferative but also anti-migratory and anti-invasion effects on HCC.

### ARG Inhibits the Activation of Wnt/β-Catenin Signaling Pathway in HCC

**Figure 3:**
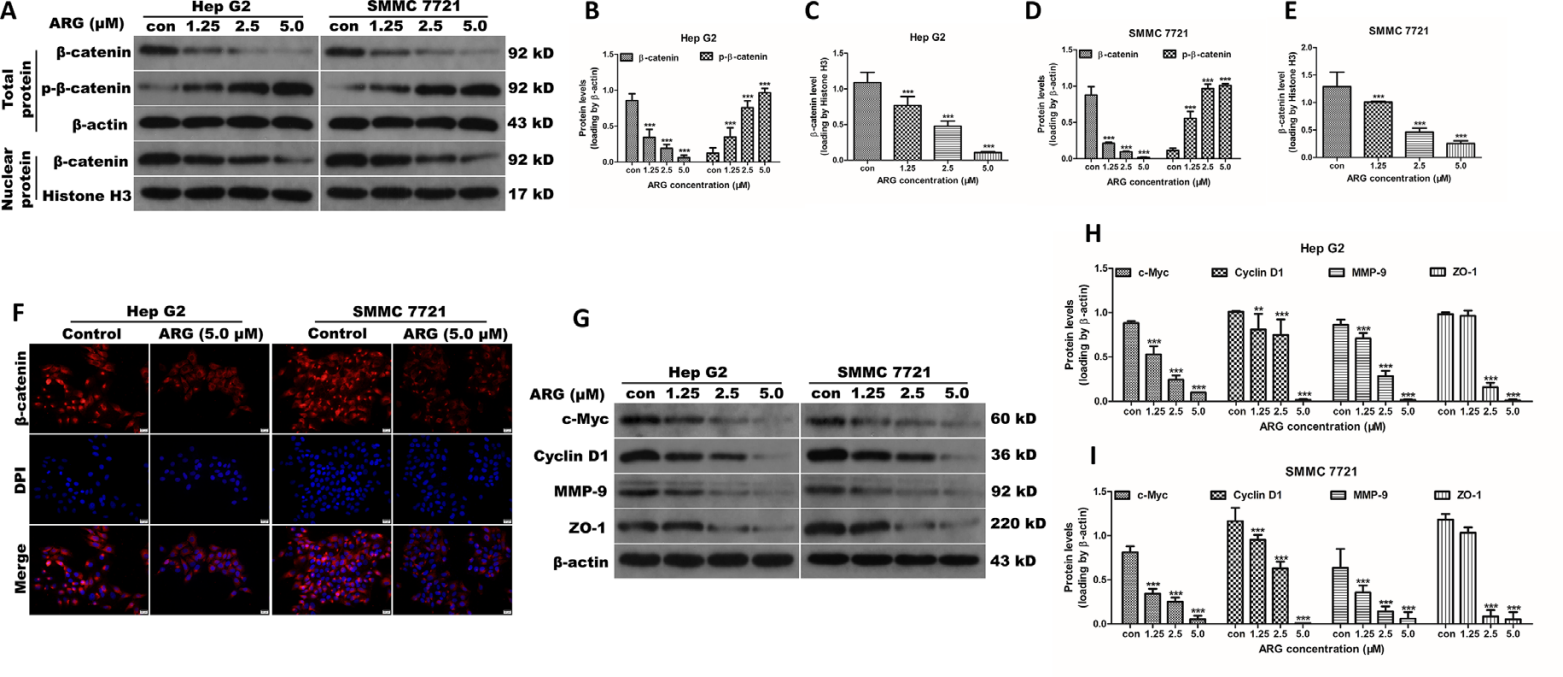
ARG suppresses the Wnt/β-catenin signaling and downregulates its downstream targets including c-myc, cyclin D1, MMP-9, and ZO-1 in HCC. **(A)** Hep G2 and SMMC 7721 cells were incubated with ARG (0, 1.25, 2.5, 5.0 μM) for 24 h, and the expression levels of β-catenin and p-β-catenin in whole cell lysate and β-catenin in nucleus were analyzed by western blotting. **(B**–**E)** Relative protein levels of β-catenin and p-β-catenin were quantified by scanning densitometry and normalized to the loading controls. **(F)** Representative images of the indirect immunofluorescence analysis of β-catenin from Hep G2 and SMMC 7721 cells treated with ARG (5.0 μM) for 24 h. Red: staining for active β-catenin, blue: nuclear staining by DAPI. **(G)** The expression levels of c-myc, cyclin D1, MMP-9, and ZO-1 in whole cell lysate were analyzed by western blotting after indicated treatments. **(H** and **I)** Relative protein levels of c-myc, cyclin D1, MMP-9, and ZO-1 were quantified by scanning densitometry and normalized to β-actin. Data were presented as means ± SD, *n* = 3, and western blotting data were representative of three independent experiments. **p < 0.01, ***p < 0.001 versus the control group.

Increasing evidence suggest that Wnt/β-catenin signaling pathway plays a key role in mediating tumor metastasis and invasion. To explore the mechanism underlying the anti-cancer effect of ARG, western blotting analysis was applied to evaluate the regulating effect of ARG on the β-catenin expression. As shown in **Figures 3A–E**, ARG treatment down-regulated the expression of β-catenin in a concentration-dependent manner in both cytoplasm and nuclear. It is considered that the phosphorylation of β-catenin and the subsequent ubiquitination/degradation are mainly responsible for attenuating the β-catenin accumulation. Therefore, we also determined the effect of ARG on the phosphorylation of β-catenin. As a result, ARG concentration-dependently led to a significant increase of phosphorylated β-catenin at Ser33/37/Thr41 residues (**Figures 3A, B, D**). The attenuated accumulation of β-catenin in both cytoplasm and nuclear was further confirmed by immunofluorescence assay as shown in **Figure 3F**. Furthermore, we also observed the effects of ARG on the transcriptional target genes of β-catenin, including ZO-1, MMP-9, c-myc, and cyclin D1. The western blotting analysis revealed that the expression of ZO-1, MMP-9, c-myc, and cyclin D1 was all down-regulated by ARG in a concentration-dependent manner (**Figures 3G–I**). The corresponding mRNA levels of ZO-1, MMP-9, c-myc, and cyclin D1 were examined by RT-qPCR and showed a similar result (**Supplementary Figure 2**). All these findings indicated that ARG inhibited the activation of Wnt/β-catenin signaling pathway in HCC.

### ARG Inhibits the Activation of Wnt/β-Catenin *via* a GSK3β-Dependent Pathway

**Figure 4:**
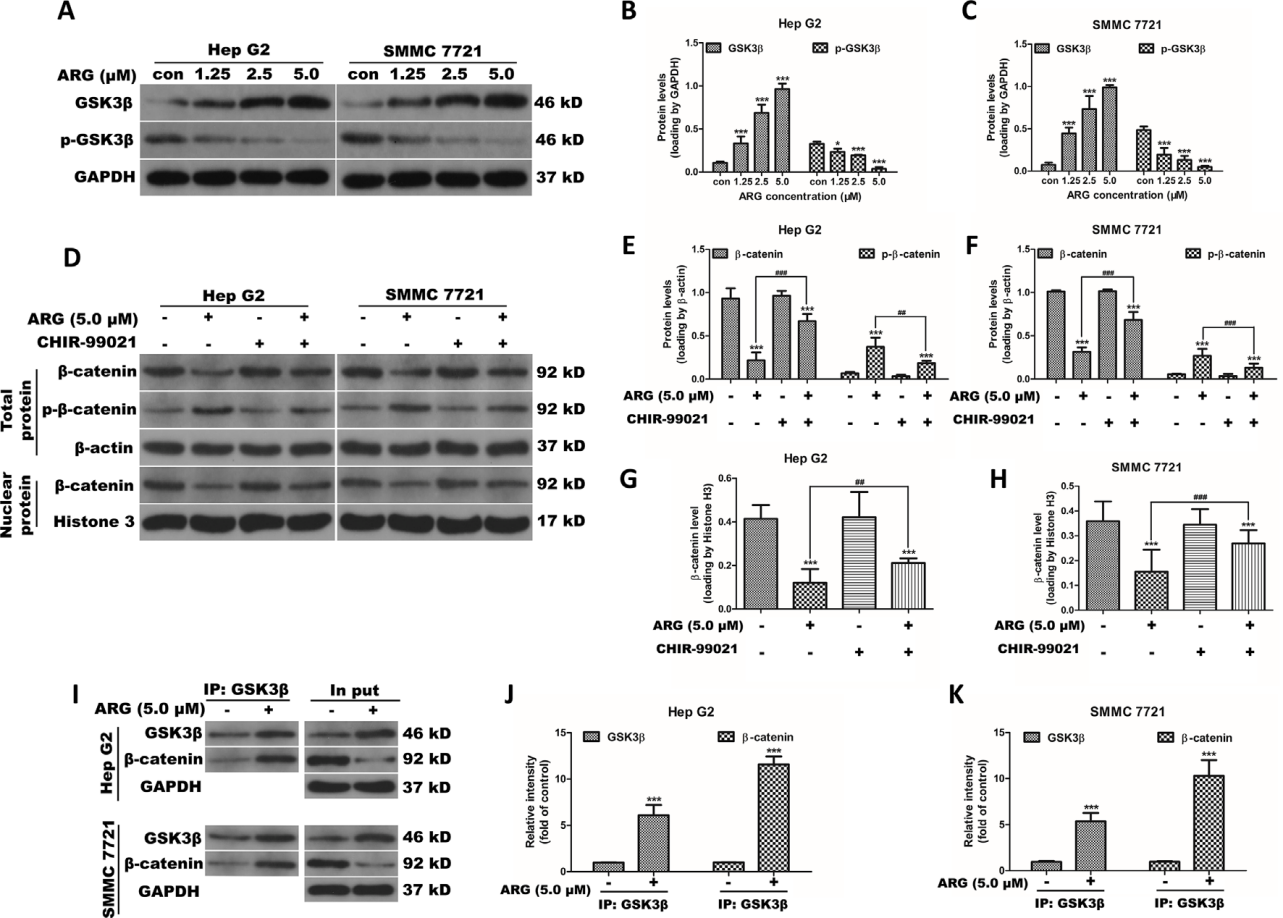
ARG inhibits the activation of Wnt/β-catenin *via* a GSK3β-dependent pathway. **(A)** Hep G2 and SMMC 7721 cells were incubated with ARG (0, 1.25, 2.5, 5.0 μM) for 24 h, and the expression levels of GSK3β and p-GSK3β in whole cell lysate were analyzed by western blotting. **(B** and **C)** Relative protein levels of GSK3β and p-GSK3β were quantified by scanning densitometry and normalized to GAPDH. Hep G2 and SMMC 7721 cells were pretreated with CHIR-99021 (5 nM) for 1 h and incubated with ARG (5.0 μM) for 24 h in the presence of CHIR-99021. **(D)** The expression levels of β-catenin and p-β-catenin in whole cell lysate and β-catenin in nucleus were analyzed by western blotting. **(E**–**H)** Relative protein levels of β-catenin and p-β-catenin were quantified by scanning densitometry and normalized to the loading controls. **(I)** Hep G2 and SMMC 7721 cells were incubated with or without ARG (5.0 μM) for 24 h, and equivalent proteins were immuno-precipitated with an anti-GSK3β antibody and visualized by western blotting analysis with GSK3β and β-catenin antibodies. **(J** and **K)** Relative protein levels of GSK3β and β-catenin were quantified by scanning densitometry. Data were presented as means ± SD, *n* = 3, and western blotting data are representative of three independent experiments. *p < 0.05, ***p < 0.001 versus the control group, ^##^p < 0.01, ^###^p < 0.001 versus the ARG group.

It is well known that the accumulation of β-catenin is regulated by a multimeric complex containing Axin, APC, and GSK3β, which induces the phosphorylation and subsequent degradation of β-catenin. To investigate the role of GSK3β in ARG-induced down-regulation of β-catenin, the total and phosphorylated GSK3β were evaluated by western blotting analysis. As shown in **Figures 4A–C**, GSK3β protein level was significantly increased in ARG-treated HCC in a concentration-dependent manner compared to the control groups, while the phosphorylation of GSK3β levels was remarkably decreased. To further confirm whether ARG inhibits Wnt/β-catenin *via* GSK3β-dependent signaling pathway, theGSK3β signal was inhibited using the specific inhibitor CHIR-99021, and the expression of β-catenin in both cytoplasm and nucleus was determined by western blotting. **Figures 4D–H** shows that GSK3β inhibitor significantly attenuated the ARG-induced depression of β-catenin signaling in HCC. In addition, Hep G2 and SMMC 7721 cells were incubated with or without ARG for 24 h, lysates were immune-precipitated with GSK3β, and western blotting analysis for β-catenin and GSK3β showed that ARG treatment enhanced of the combination between GSK3β and β-catenin (**Figures 4I–K**). Altogether, these findings suggested that GSK3β might be involved in ARG-induced inhibition of Wnt/β-catenin in HCC.

### ARG Exerts Anti-Metastasis and Anti-Invasion Effects by Altering the EMT-Related Proteins in HCC

**Figure 5:**
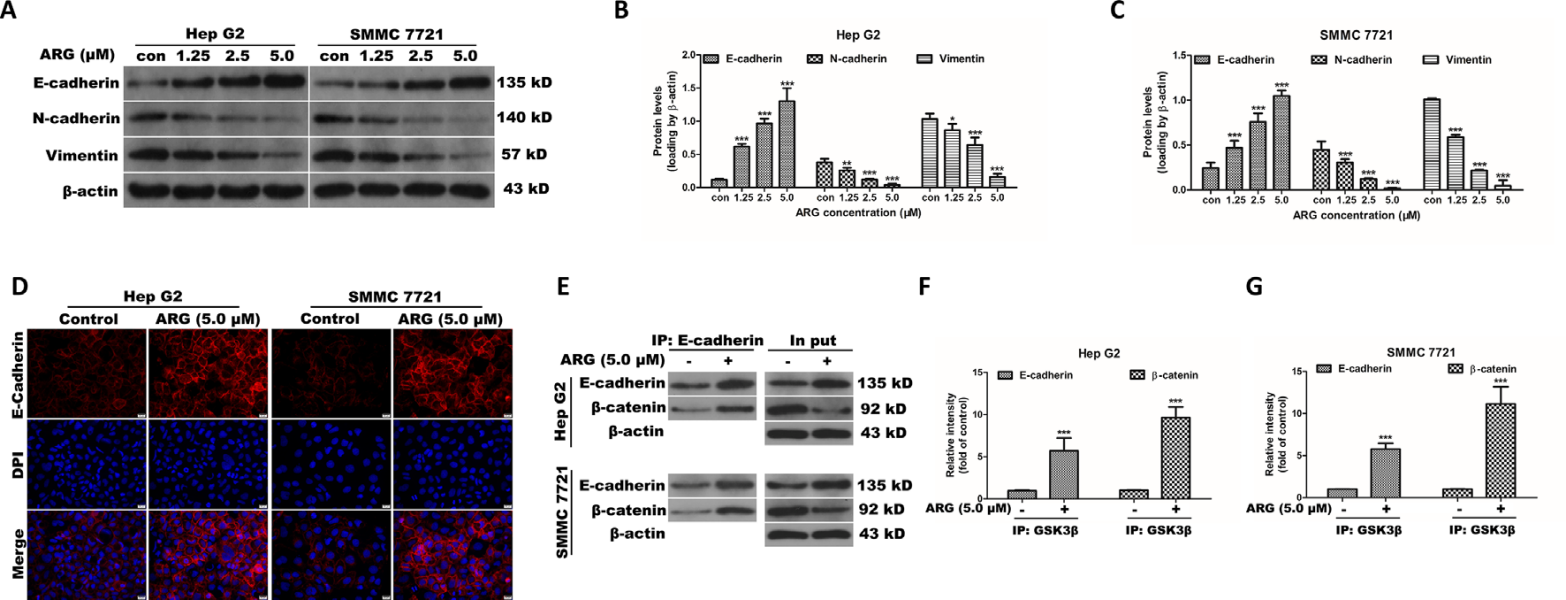
ARG exerts anti-metastasis and anti-invasion effects by altering the EMT-related proteins in HCC. **(A)** Hep G2 and SMMC 7721 cells were incubated with ARG (0, 1.25, 2.5, 5.0 μM) for 24 h, and the expression levels of E-cadherin, N-cadherin, and vimentin in whole cell lysate were analyzed by western blotting. **(B** and **C)** Relative protein levels of E-cadherin, N-cadherin, and vimentin were quantified by scanning densitometry and normalized to β-actin. **(D)** Representative images of the indirect immunofluorescence analysis of E-cadherin from Hep G2 and SMMC 7721 cells treated with ARG (5.0 μM) for 24 h. Red: staining for E-cadherin, blue: nuclear staining by DAPI. **(E)** Hep G2 and SMMC 7721 cells were incubated with or without ARG (5.0 μM) for 24 h, and equivalent proteins were immuno-precipitated with an E-cadherin antibody and visualized by western blotting analysis with E-cadherin and β-catenin antibodies. **(F** and **G)** Relative protein levels of E-cadherin and β-catenin were quantified by scanning densitometry. Data were presented as means ± SD, *n* = 3, and western blotting data are representative of three independent experiments. *p < 0.05, **p < 0.01, ***p < 0.001 versus the control group.

Epithelial–mesenchymal transition (EMT) is an important developmental process in tumor migration and invasion. EMT markers including E-cadherin, N-cadherin, and vimentin were examined the in HCC after ARG treatment by western blotting. The epithelial marker E-cadherin was found concentration-dependently increased in the ARG treated groups. Conversely, the mesenchymal markers N-cadherin and vimentin were dose-dependently decreased (**Figures 5A–C**). The corresponding mRNA levels of E-cadherin, N-cadherin, and vimentin were examined by RT-qPCR and showed a similar result (**Supplementary Figure 3**). In addition, E-cadherin expression was visualized by immunofluorescence assay. We could also observe that ARG resulted in an up-regulation of E-cadherin, as shown in **Figure 5D**. To further investigate whether Wnt/β-catenin depression was related to E-cadherin, HCC lysates were immune-precipitated with E-cadherin, and then western blotting was used for β-catenin and E-cadherin detection. As shown in **Figures 5E–G**, treatment of ARG significantly intensified the combination between E-cadherin and β-catenin in both Hep G2 and SMMC 7721 cells. Therefore, these results demonstrated that the anti-metastasis and anti-invasion effects of ARG were related to the restoration of the EMT process.

### ARG Exerts Anti-Metastasis and Anti-Invasion Effects *In Vivo*

**Figure 6:**
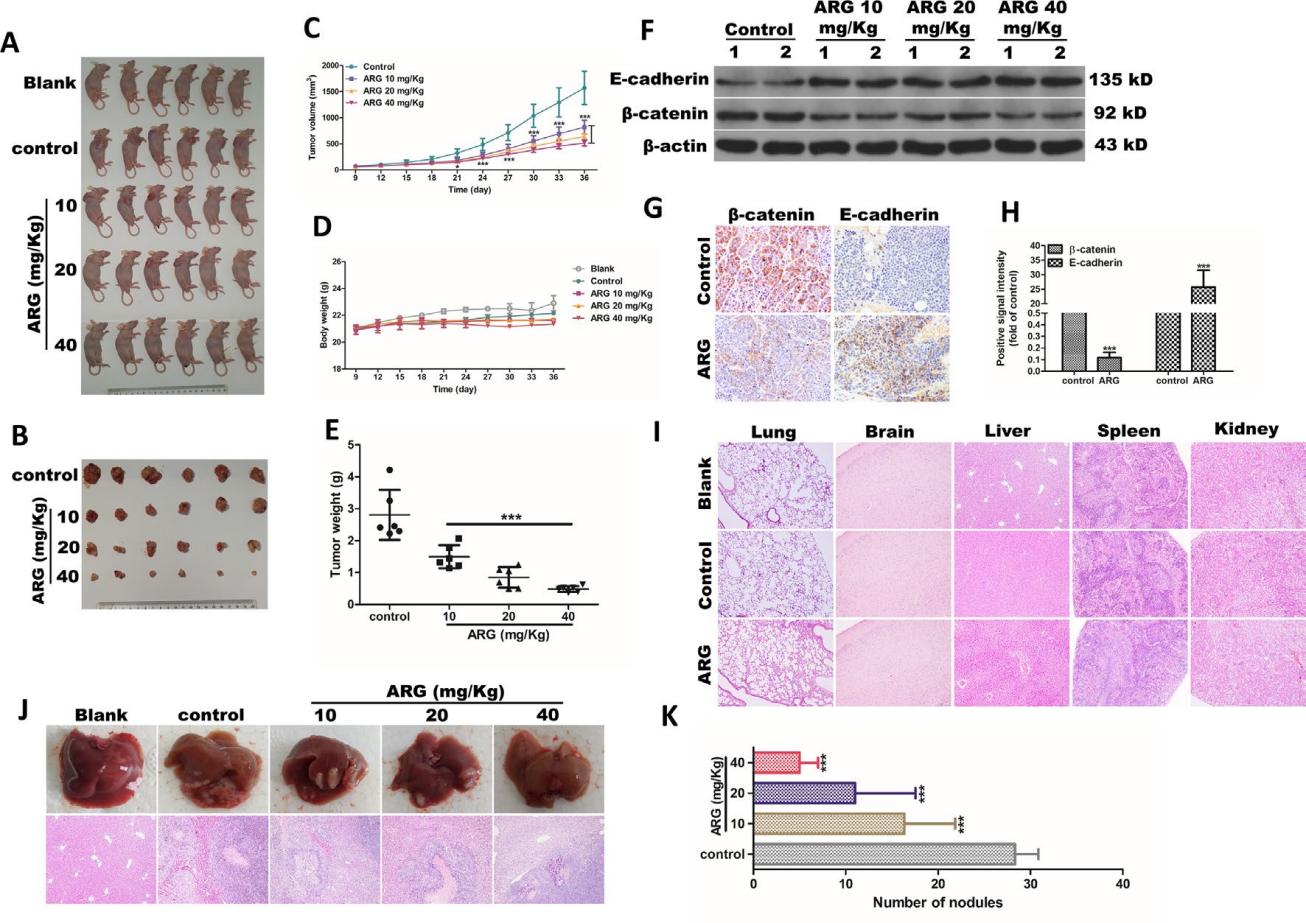
ARG exerts anti-metastasis and anti-EMT effects *in vivo*. Athymic nu/nu mice were subcutaneously transplanted with 2×10^6^ cells of Hep G2 cells into the axillary fossa to build a xenograft tumor model. The mice were divided into five groups (*n* = 6) including blank, control, and three ARG (10, 20, 40 mg/kg) administered groups. **(A)** All the mice were sacrificed by cervical dislocation. **(B)** Six subcutaneous tumors of each group were shown at day 36. **(C)** The tumor volumes and **(D)** body weight were measured every 3 days. **(E)** Mean of tumor weight in control and ATG-treated groups. **(F)** The protein levels of E-cadherin and β-catenin in tumor tissues were detected by western blotting. **(G** and **H)** IHC assay revealing the E-cadherin and β-catenin protein levels from the tumor tissues in control and ARG-treated (40 mg/kg) groups. **(I)** Tissue sections of lungs, brain, liver, spleen, and kidneys from blank, control, and ARG-treated (40 mg/kg) groups were determined by hematoxylin and eosin stain (100× magnification). Athymic nu/nu mice were intravenously transplanted with 1×10^5^ cells of Hep G2 cells into the tail vein, and the grouping and the ARG administration were similar to xenograft tumor models. **(J** and **K)** Livers were excised to compare the pattern of hepatic tumor nodule formation among the experimental groups. Data were presented as means ± SD, *n* = 6. Western blotting, HE, and IHC data were representative of three independent experiments. *p < 0.05, ***p < 0.001 versus the control group.

Our study also investigated whether ARG could ameliorate the tumor growth and metastasis of HCC *in vivo*. As shown in **Figures 6A, B**, the growth of subcutaneous transplantation tumors was dramatically inhibited compared to the control group after 5 weeks of ARG administration. The volume of the tumors, which were determined every 3 days, showed that ARG significantly inhibited the tumor growth since the 21st day (**Figure 6C**). The tumor weight analysis indicated that the inhibitory effect of ARG gradually enhanced with the dosage increase (**Figure 6E**). Meanwhile, mean body weight (**Figure 6D**) and tissue sections including lungs, brains, livers, spleens, and kidneys (**Figure 6I**) did not show any significant differences in blank, control, or ARG-treated mice. In the metastatic tumor models, the numbers of tumor nodules in livers were decreased by the ARG treatment (**Figures 6J, K**). To gain insight to the mechanism of ARG in inhibiting tumor metastasis *in vivo*, we harvested the Hep G2 tumor xenografts. Proteins were extracted to assess the levels of E-cadherin and β-catenin. As shown in **Figure 6F**, up-regulating E-cadherin and down-regulating β-catenin levels were observed in tumors from the ARG-treated groups, which was further confirmed by IHC assay (**Figures 6G, H**). The regulatory effects of ARG in E-cadherin and β-catenin *in vivo* were consistent with that *in vitro*.

## Discussion

Tumor metastasis is considered as a principal reason in death of patients who suffered from hepatocellular carcinoma (Mehlen and Puisieux, 2006). Accumulating evidence suggests that tumor metastasis is crucially dependent on the epithelial to mesenchymal transition (EMT) of tumor cells (Li and Li, 2015). Therefore, it is of great therapeutic benefit to develop effective therapeutic strategies targeting on EMT in hepatocellular carcinoma to prevent malignant metastasis (Amawi et al., 2017). Natural products are major sources providing promising leads for the development of novel anti-cancer drugs due to their potentially low toxicity and potential effectiveness. We have previously demonstrated that ARG could induce apoptosis in HCC including Hep G2 and SMMC7721 cells with mild cytotoxicity in normal hepatic cells (Lu et al., 2015); however, whether the anti-EMT and anti-metastasis were also involved in the anti-tumor activity of ARG in hepatocellular carcinoma still remained unclear.

In the present study, we incubated Hep G2 and SMMC 721 with ARG at a lower concentration compared to our former research. Although ARG still significantly inhibited the proliferation in a concentration- and time-dependent manner, most Hep G2 and SMMC 7721 remained alive. We mostly focused on the effect of ARG on the surviving cells. As we observed, the migration and invasion abilities of Hep G2 and SMMC 7721 were significantly inhibited, accompanied by the attenuation of Wnt/β-catenin signaling and its target genes including ZO-1, MMP-9, c-myc, and cyclin D1. It has been proven that the dysregulation of Wnt/β-catenin signaling played an important role in promoting migration and invasion in various human cancers, including hepatocellular carcinoma (Chen et al., 2017). Repressing the activation of Wnt/β-catenin signaling by natural compounds or plant extracts has been reported in several studies, leading to a restraint of migration and invasion in malignant tumors. Huynh et al. demonstrated destruxin B reducing the aggressiveness and invasive potential of hepatocellular carcinoma cell (HCC) *via* inhibiting Wnt/β-catenin activation, and its combination with sorafenib achieved a better management in metastatic HCC (Huynh et al., 2014). Zhang et al. revealed that ARG inhibited angiogenesis and HCT116 cell migration and invasion through down-regulating the H1F4A and Wnt/β-catenin (Zhang et al., 2015). Additionally, Wei et al. indicated that the aberrant activation of Wnt/β-catenin caused by nicotine was suppressed by nuciferine in human lung adenocarcinoma epithelial cells, showing a weaker potential to migration and invasion (Liu et al., 2015a). Obviously, these findings were all consistent with our data, which proved Wnt/β-catenin a target of ARG in reducing the aggressiveness and invasive potential of hepatocellular carcinoma.

Furthermore, we also observed that ARG significantly increased the accumulation of GSK3β protein and decreased GSK3β phosphorylation, accompanied by the combination enhancement between GSK3β and β-catenin. Increasing evidence indicated that GSK3β was one of the pivotal up-stream components of β-catenin signaling, which included GSK3β, APC, and Axin, regulating the phosphorylation of β-catenin in the cytoplasm and targeting it for degradation (Matsubayashi et al., 2004; Kikuchi et al., 2006). Accumulated GSK3β was able to facilitate ubiquitin/proteasome dependent degradation of β-catenin resulting in the transduction signal attenuation (Sarkar et al., 2010). Hseu et al. reported that antrodia camphorata GSK3β-dependently decreased nuclear translocation of Wnt/β-catenin in SW620claudin1+ cells, which exhibited an anti-metastasis effect on human colon cancer cells (Hseu et al., 2017). Reversely, the repression of GSK3β might intensify Wnt/β-catenin activation, promoting tumor growth and metastasis. lncRNA small nucleolar RNA host gene 5 (SNHG5) was found acting as a competing endogenous RNA competitively binding with miR-26a-5p and thereby modulating the de-repression of downstream target GSK3β, functionally promoted tumor metastasis and induced EMT via activating Wnt/β-catenin pathway (Li et al., 2018). However, GSK3β was not the only up-stream regulator of β-catenin. It was revealed that CoQ0, a major active constituent of antrodia camphorata, inhibited the migration and invasion of melanoma cells via down-regulating Wnt/β-catenin mainly owing to the increasing of Axin rather than GSK3β (Hseu et al., 2016). Therefore, we used the specific phosphorylation inhibitor of GSK3β to interfere the GSK3β dependent degradation of β-catenin. The results confirmed that ARG attenuated the activation of Wnt/β-catenin through GSK3β-dependent pathway.

Tumor metastasis has been demonstrated to be a complex process involving cell migration, invasion, growth, adhesion, and proteolytic degradation of tissue barriers such as the extracellular matrix (ECM) and basement membrane (Mehlen and Puisieux, 2006). Tumor cells might not be capable to invade or migrate to other tissues until EMT happened, with the hallmarks of cell–cell adhesion loss, cell polarity decrease, cytoskeleton reorganization, and acquisition of increased migratory characteristics (Tsai and Yang, 2013; Lamouille et al., 2014). Tight junction proteins including ZO-1 functioned as complete barriers between epithelial and endothelial cells contributing to maintain cell polarity (Tabasum and Singh, 2019). Our data showed a dramatically decrease ZO-1 in HCC after ARG administration. Matrix metalloproteinases (MMPs), a family of structurally and functionally related zinc-dependent enzymes, have been revealed to promote the proteolytic degradation of ECM components (Zheng et al., 2006; Bae et al., 2016). Our data also showed a down-regulation in MMP-9, which might contribute to the degradation of ECM proteins in HCC. Similar results were represented by Lou et al., which proved that ARG significantly inhibited the migration and invasion of MDA-MB-231 cells by downregulating the expression of MMP-2, MMP-9, and Heparanase (Lou et al., 2017). These findings indicated that ARG acted as an anti-metastatic substance against HCC by modulating the possible molecules involving ZO-1 and MMP-9.

E-cadherin, a Ca2+-dependent transmembrane glycol-protein, has been considered as an “invasion suppressor,” playing an important role in maintaining intercellular adhesion through the formation of adherent junctions (Mareel et al., 1994; Gumbiner, 2000). It has been proved that E-cadherin/β-catenin complex regulated the adhesion of tumor cell through attaching to actin cytoskeleton (Tian et al., 2011) and contributed to the stabilization of β-catenin via restricting its translocation to nucleus (Orsulic et al., 1999). Much attention has been paid into modulating tumor metastasis via preventing the combination between β-catenin and E-cadherin to maintain cell–cell adhesion and to inhibit β-catenin translocating into the nucleus to act as the transcription effector. Yo-Han Han et al. previously found that ARG controlled EMT in CT26 cells through increasing the transcription of epithelial marker E-cadherin and decreasing the transcription of mesenchymal markers including N-cadherin, vimentin, β-catenin, and Snail (Han et al., 2016). In our study, ARG induced a dramatic increase in the expression of E-cadherin and restored E-cadherin/β-catenin complex formation in HCC. This data supported that ARG exhibited an anti-EMT effect on hepatocellular carcinoma related to the regulation of E-cadherin/β-catenin complex formation. In addition to E-cadherin, the expression of mesenchymal markers including N-cadherin and vimentin were repressed, further indicating that ARG was able to elevate HCC adhesion and impeded the metastasis.

In vivo, intraperitoneal injection of ARG not only significantly inhibited the growth of subcutaneous transplantation tumor but also dramatically alleviated the tumor metastasis in liver, without any toxicity. Especially, the E-cadherin up-regulation and β-catenin down-regulation were also found in tumor xenograft nude mice, which was accordant with the data in vitro. Our data demonstrated that ARG exerted anti-EMT and anti-metastasis activities against hepatocellular carcinoma, which might make it a candidate as a preventive agent for cancer metastasis.

## Conclusion

**Figure 7:**
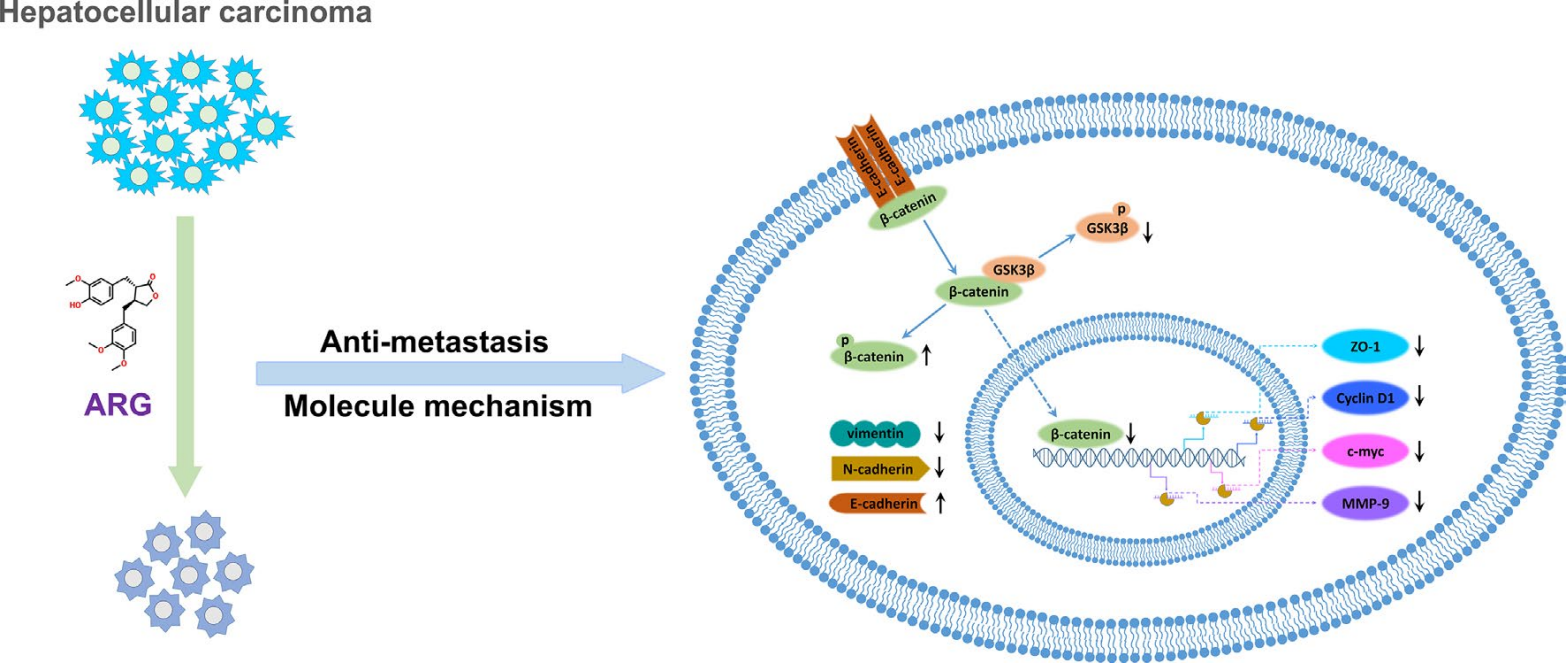
The molecular mechanism describing the attenuation effect of ARG in hepatocellular carcinoma metastasis.

In conclusion, our observations indicated that ARG exerted an inhibitory effect on hepatocellular carcinoma metastasis in vitro and in vivo and improved the current understanding of ARG anti-tumor activity against hepatocellular carcinoma. Furthermore, we firstly revealed that ARG attenuated the migration and invasion of HCC via suppressing GSK3β-dependent Wnt/β-catenin signaling pathway. Additionally, the regulation of the expression levels of EMT-related proteins including ZO-1, MMP-9, E-cadherin, N-cadherin, and vimentin was also involved in the molecular mechanisms (**Figure 7**). We provided clear evidence that ARG might be a promising candidate as a preventive agent for cancer metastasis.

## Data Availability

Publicly available datasets were analyzed in this study. This data can be found here: https://pan.baidu.com/s/1XEzLv7CkSNBNySm4Qy3tIA

## Ethics Statement

This study was carried out in accordance with the principles and procedures of the Institute Ethical Committee for Experimental Use of Animals in Northwest A&F University. This study was approved by the Institute Ethical Committee for Experimental Use of Animals in Northwest A&F University (Permit Number: 20181109).

## Author Contributions

ZL and LC developed the original idea, designed the experiments, and elaborated data, contributing equally to this work. TM, LC, and ZL performed experiments and prepared figures. XL and HZ edited and reviewed the final version of the article. YL and DT supervised the study. All listed authors contributed to article writing.

## Funding

This work was supported by the National Natural Science Foundation of China (31602105) and the Ph.D. Star-up Fund of Northwest A&F University (Z109021610).

## Conflicts of Interest Statement

The authors declare that the research was conducted in the absence of any commercial or financial relationships that could be continued as a potential conflict of interest.

## Abbreviations

ARG, Arctigenin; HCC, hepatocellular carcinoma; EMT, epithelial-mesenchymal transition; Fz, Frizzled; LRP, Low-density lipoprotein receptor-related protein; DPI , diphenylene iodide; CK1a, casein kinase 1a; APC, adenomatous polyposis coli; GSK3ß , glycogen synthase kinase 3ß; MMP, Matrix metalloproteinases; FBS, fetal bovine serum; DMSO, Dimethyl sulfoxide; IHC, immunohistochemistry; HE, hematoxylin-eosin; ANOVA, one-way analysis of variance; ECM, extracellular matrix.

## Supplementary Material

Supplementary Figure 1. Effect of Arctigenin (ARG) on normal liver cell line (LO2). LO2 cells were incubated with ARG (0,1.25, 2.5, 5.0 μM) for 24 and 48 h, respectively. Cell viability was determined by MTT assay. Results were presented as mean ± SD, n = 3.

Supplementary Figure 2. Effect of ARG on ZO-1, MMP-9, c-myc and Cyclin D1 mRNA expression level. Hep G2 and SMMC 7721 cells were treated with ARG (0, 1.25, 2.5, 5.0 μM) for 24 h. Total RNA were prepared for analyzing the mRNA expression level of ZO-1, MMP-9, c-myc and Cyclin D1 by qRT-PCR using specific primers. Data were presented as mean ± SD, n = 3. *p < 0.05, **p < 0.01, ***p < 0.001 versus the control group.

Supplementary Figure 3. Effect of ARG on E-cadherin, N-cadherin and vimentin mRNA expression level. Hep G2 and SMMC 7721 cells were treated with ARG (0, 1.25, 2.5, 5.0 μM) for 24 h. Total RNA were prepared for analyzing the mRNA expression level of E-cadherin, N-cadherin and vimentin by qRT-PCR using specific primers. Data were presented as mean ± SD, n = 3. *p < 0.05, **p < 0.01, ***p < 0.001 versus the control group.
