# Supplementary figures and images for: Arctigenin Attenuates Tumor Metastasis Through Inhibiting Epithelial–Mesenchymal Transition in Hepatocellular Carcinoma via Suppressing GSK3β-Dependent Wnt/β-Catenin Signaling Pathway In Vivo and In Vitro
Source: Front Pharmacol. 2019 Aug 29;10:937. doi: 10.3389/fphar.2019.00937 (PMC6726742; doi:10.3389/fphar.2019.00937)

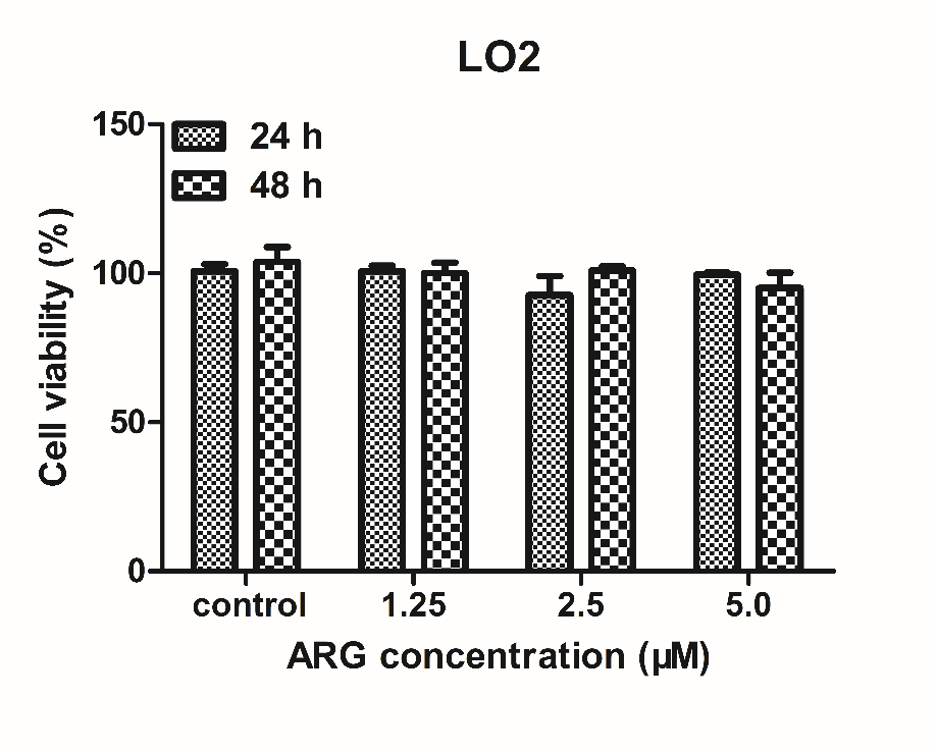

Supplement: Supplementary Figure 1. — Effect of Arctigenin (ARG) on normal liver cell line (LO2). LO2 cells were incubated with ARG (0,1.25, 2.5, 5.0 μM) for 24 and 48 h, respectively. Cell viability was determined by MTT assay. Results were presented as mean ± SD, n = 3. [file Image_1.tif]

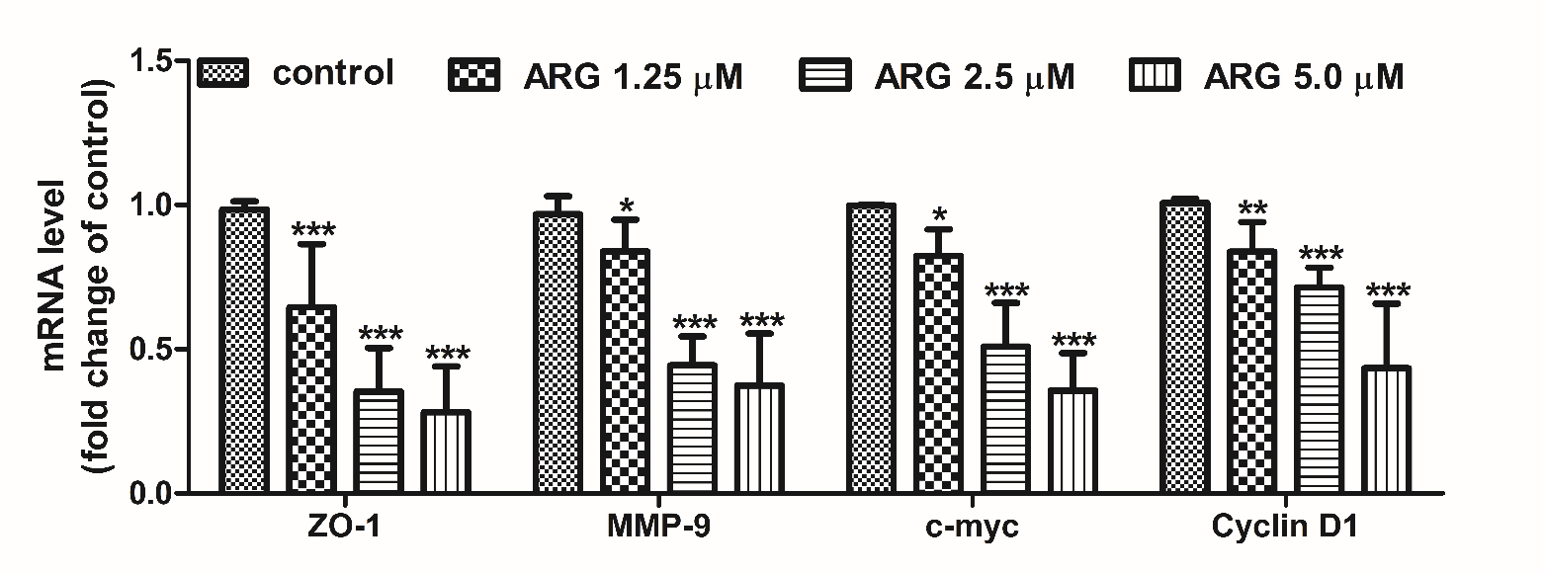

Supplement: Supplementary Figure 2. — Effect of ARG on ZO-1, MMP-9, c-myc and Cyclin D1 mRNA expression level. Hep G2 and SMMC 7721 cells were treated with ARG (0, 1.25, 2.5, 5.0 μM) for 24 h. Total RNA were prepared for analyzing the mRNA expression level of ZO-1, MMP-9, c-myc and Cyclin D1 by qRT-PCR using specific primers. Data were presented as mean ± SD, n = 3. *p < 0.05, **p < 0.01, ***p < 0.001 versus the control group. [file Image_2.tif]

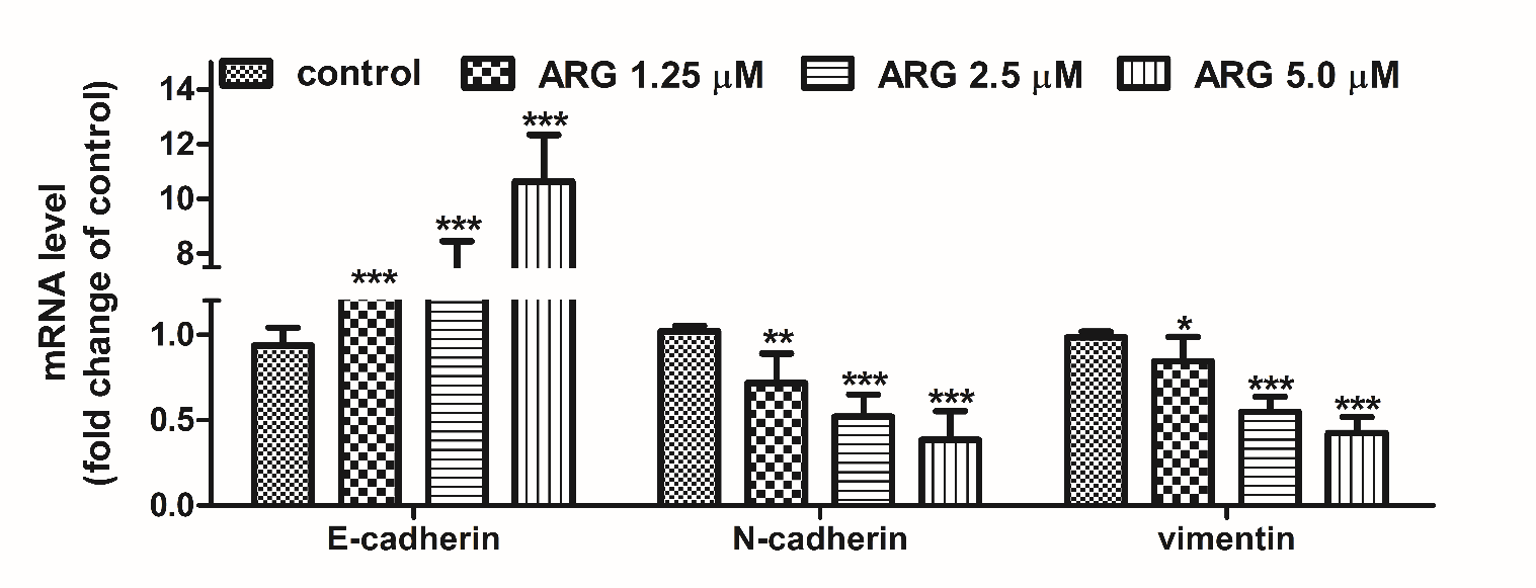

Supplement: Supplementary Figure 3. — Effect of ARG on E-cadherin, N-cadherin and vimentin mRNA expression level. Hep G2 and SMMC 7721 cells were treated with ARG (0, 1.25, 2.5, 5.0 μM) for 24 h. Total RNA were prepared for analyzing the mRNA expression level of E-cadherin, N-cadherin and vimentin by qRT-PCR using specific primers. Data were presented as mean ± SD, n = 3. *p < 0.05, **p < 0.01, ***p < 0.001 versus the control group. [file Image_3.tif]
