# Supplementary material for: Arctigenin Attenuates Tumor Metastasis Through Inhibiting Epithelial–Mesenchymal Transition in Hepatocellular Carcinoma via Suppressing GSK3β-Dependent Wnt/β-Catenin Signaling Pathway In Vivo and In Vitro
Source: Front Pharmacol. 2019 Aug 29;10:937. doi: 10.3389/fphar.2019.00937 (PMC6726742; doi:10.3389/fphar.2019.00937)
Supplement: Supplementary file 4 [file Table_1.docx]

**Supplementary Table 1.** Sequences for real-time RT-qPCR primers

| **Gene** | **Forward (5*’*-3*’*)** | **Reverse (5’-3’)** |
| --- | --- | --- |
| MMP-9 | AGACCAAGGGTACAGCCTGTTC | GGCACGCTGGAATGATCTAAG |
| ZO-1 | AATTGCTCGTATGGATCC | GCCTGCAATGCGCTTGAA |
| E-cadherin | AATGGCGGCAATGCAATCCCAAGA | TGCCACAGACCGATTGTGGAGATA |
| N-cadherin | TGGAGAACCCCATTGACATT | TGATCCCTCAGGAACTGTCC |
| Vimentin | CGGAAAGTGGAATCCTTGCA | CACATCGATCTGGACATGCTG |
| c-myc | AATGGCAATGGCAATCTGGC | CCTAGCGGTCGCCTTAAATGA |
| Cyclin D1 | GGATCCGTAGTCGAAGC | CCATCGTTCAGATGCCG |
| β-actin | CGGTAACGTTCGAAGTTAC | ATTCGTTGCCAGTAACGTC |
